# Neuro‐associated SEMA3C^+^ tumour cells promote gastric cancer peritoneal metastasis via EBF2–FGF7–FGFR2‐mediated tumour–endothelial crosstalk

**DOI:** 10.1002/ctm2.70861

**Published:** 2026-10-15

**Authors:** Yajing Zhou, Chao Dong, Weikang Li, Minghao Sun, Qingyu Xie, Lixing Gu, Pengbo Wang, Chengxiang Xu, Tianzhan Gao, Caixin Zhu, Kaipeng Duan, Jin Zhou, Dongbao Li

**Affiliations:** ^1^ Department of General Surgery The First Affiliated Hospital of Soochow University Suzhou Jiangsu Province China

**Keywords:** FGF–FGFR signalling pathway, gastric cancer peritoneal metastasis, organoid–endothelial cell co‐culture, semaphorin, tumour innervation

## Abstract

**Background:**

Recent studies have established that the nervous system is not merely a bystander in tumour progression but can actively remodel the tumour microenvironment through the neuro–tumour axis, thereby promoting tumour invasion, metastasis and survival. Semaphorin 3C (SEMA3C) has been reported to regulate tumour migration, angiogenesis and immune suppression, highlighting its potential role in tumour progression. Although the neuro–tumour axis has been identified in multiple cancer types, its role and underlying mechanisms in gastric cancer peritoneal metastasis (GCPM) remain unclear.

**Methods:**

We performed single‐nucleus RNA sequencing (snRNA‐seq) of GCPM tissues to profile tumour cell heterogeneity and identify tumour cell populations associated with neural‐related signalling. Candidate tumour–microenvironmental interactions were further investigated using patient‐derived organoids and co‐culture models, and the identified signalling axis was validated using corresponding in vivo models.

**Results:**

SnRNA‐seq identified a distinct subpopulation of poorly differentiated tumour cells with pronounced stem‐like properties and high SEMA3C expression. This subpopulation was associated with neural‐related features and interacted with tumour‐associated endothelial cells, thereby contributing to remodelling of the metastatic microenvironment. Furthermore, these cells enhanced tumour–endothelial communication by activating the fibroblast growth factor receptor‐associated signalling pathway. The transcription factor early B‐cell factor 2 (EBF2) regulates the expression of FGF7 within this subpopulation, thereby strengthening FGF7–FGFR2 signalling and promoting tumour progression. Functional validation further demonstrated that SEMA3C depletion impaired organoid growth and tumour–endothelial interactions, whereas disruption of the SEMA3C–EBF2–FGF7–FGFR2 signalling axis attenuated tumour growth and peritoneal tumour burden in vivo. Additional experimental validation supported the potential involvement of neural‐related signalling in the SEMA3C‐associated tumour cell state.

**Conclusions:**

Our findings identify a SEMA3C^+^ tumour cell subpopulation associated with neural‐related signalling and suggest that SEMA3C promotes tumour–endothelial interactions through the EBF2–FGF7–FGFR2 signalling axis, providing a potential mechanistic link between neural regulation and microenvironmental remodelling in peritoneal metastasis of gastric cancer.

## Key points

- Single‐nucleus RNA sequencing identifies a SEMA3C^+^ tumour cell subpopulation with stem‐like properties and neural‐related features in gastric cancer peritoneal metastasis.
- SEMA3C enhances tumour‐endothelial crosstalk through the EBF2‐FGF7‐FGFR2 signalling axis.
- Disruption of the SEMA3C‐EBF2‐FGF7‐FGFR2 signalling axis attenuates tumour growth and reduces peritoneal tumour burden in vivo.

## Graphical abstract

Single‐nucleus sequencing identified a semaphorin 3C (SEMA3C)‐high stem‐like tumour subpopulation that drives gastric cancer peritoneal metastasis through early B‐cell factor 2 (EBF2)‐mediated FGF7 expression and enhanced FGF7–FGFR2 signalling with endothelial cells. Targeting the FGF–FGFR pathway may provide a promising therapeutic strategy.

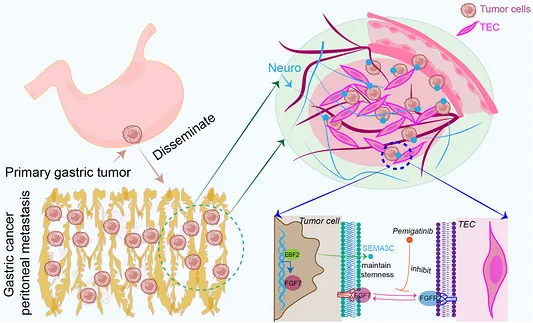

## 1 INTRODUCTION

Recent studies have illustrated that the nervous system plays a critical role in tumour progression, functioning not only as a passive bystander but also as an active promoter of malignant development through structural and functional integration. Both clinical observations and basic research have shown that nerve fibre density within tumour tissues is positively correlated with tumour invasiveness. In brain metastases, tumour cells and neurons can form bona fide synaptic structures, establishing electrophysiological coupling that directly regulates tumour cell proliferation and migration.^1^ Tumours are capable of actively remodelling the local neural microenvironment. By secreting neurotrophic factors, such as nerve growth factor (NGF), brain‐derived neurotrophic factor (BDNF) and GDNF, as well as axon guidance molecules, tumour cells can induce neurogenesis and axonal growth, alter nerve trajectories and increase nerve fibre density.^2^ Highly metastatic tumours exhibit significantly greater nerve density compared to those with low metastatic potential, suggesting that tumours gain a progression advantage through neural microenvironment remodelling. Beyond brain tumours, peripheral tumours can also recruit new nerve fibres via axonogenesis, thereby enhancing local neural innervation and establishing electrical or metabolic networks similar to those observed in brain tumours, which provide functional support for tumour survival and metastasis.^3^, ^4^ Moreover, tumours can precisely regulate the spatial distribution of nerve fibres by secreting axon‐inhibitory factors, thereby further optimising the neural microenvironment in a manner that favours tumour growth and invasion.^5^, ^6^ Collectively, these findings highlight the central role of the neuro–tumour axis in tumour progression and support potential therapeutic strategies targeting neurogenesis and axon guidance signalling pathways, offering new insights into the complex regulation of the tumour microenvironment.

The semaphorin protein family comprises a group of highly conserved extracellular signalling molecules that play multifaceted roles in neurodevelopment, angiogenesis, immune regulation and tumour biology.^7^, ^8^, ^9^ The formation of the neuro–tumour axis is closely associated with semaphorin signalling, which plays a role in regulating tumour cell proliferation, migration, invasion and the immune microenvironment, thereby forming a multidimensional regulatory network. Semaphorins can be broadly classified into secreted types (SEMA2, SEMA3 and SEMA5 subclasses) and membrane‐bound types (SEMA1, SEMA4, SEMA6 and SEMA7 subclasses), and they primarily signal through plexin and neuropilin receptors to regulate the functions of neurons, endothelial cells and immune cells.^10^ Within the tumour microenvironment, semaphorins can inhibit infiltration of CD8^+^ T cell while promoting the expansion of immunosuppressive cell populations, thereby facilitating immune evasion. Monoclonal antibodies targeting semaphorin pathways have been shown to enhance T‐cell infiltration and activation; when combined with immune checkpoint inhibitors, they can further remodel the tumour microenvironment, underscoring their therapeutic potential.^11^ Among the semaphorin family, class 3 semaphorins, as secreted molecules, regulate tumour cell migration, angiogenesis and the immune microenvironment via Neuropilin/Plexin receptors. Notably, semaphorin 3C (SEMA3C) can signal through NP1/NP2 and Plexin receptors to activate ERK signalling, thereby promoting epithelial–mesenchymal transition (EMT), autophagy and PD‐L1 upregulation, ultimately enhancing cancer cell invasiveness and immunosuppression.^12^

Peritoneal metastasis of gastric cancer represents the most common form of advanced disease dissemination and is characterised by high heterogeneity, as well as pronounced invasive and metastatic potential. However, the molecular basis underlying these effects has yet to be fully elucidated, and comprehensively deciphering the complex interactions between tumour cells and the microenvironment continues to be a major challenge. In recent years, advances in single‐nucleus transcriptomic technologies have enabled high‐resolution characterisation of cellular heterogeneity, population dynamics and tumour–microenvironment interactions in peritoneal metastases.^13^, ^14^, ^15^ Patient‐derived organoid (PDO) models, which preserve the genomic and phenotypic characteristics of primary or metastatic tumours, can be further co‐cultured with endothelial cells, immune cells and other microenvironmental components. This approach facilitates systematic investigation of tumour signalling networks and intercellular functional interactions.^16^ Meanwhile, patient‐derived xenograft (PDX) and patient‐derived organoid xenograft (PDOX) models can recapitulate the in situ microenvironment of peritoneal metastases in vivo, providing robust platforms for studying tumour biology and evaluating therapeutic efficacy.^17^, ^18^ The integration of single‐nucleus analyses, organoid systems and in vivo xenograft models enables a multidimensional dissection of the molecular mechanisms underlying gastric cancer peritoneal metastasis (GCPM), thereby offering a strong experimental foundation for identifying potential therapeutic targets and advancing precision treatment strategies. However, it remains unclear whether specific tumour cell states within GCPMs are associated with neural signalling and contribute to metastatic progression. Given the marked cellular heterogeneity of peritoneal metastases, we therefore used single‐nucleus RNA sequencing (snRNA‐seq) to identify neural‐related tumour cell states, followed by PDO and tumour–endothelial co‐culture models and in vivo xenograft models for functional validation. Based on the established roles of semaphorin signalling in axon guidance and tumour–microenvironment regulation, we further focused on SEMA3C as a potential link between neural‐related tumour cell states and microenvironmental remodelling.

In this study, we performed snRNA‐seq of GCPMs and identified a distinct subpopulation of poorly differentiated tumour cells with pronounced stem‐like properties that secrete the axon‐repelling factor SEMA3C. This subpopulation is capable of remodelling the metastatic microenvironment through interactions with tumour‐associated endothelial cells (TECs), thereby reshaping the metastatic niche and regulating the tumour neuroendocrine landscape. Furthermore, these cells enhance tumour–endothelial communication by activating the FGF–FGFR signalling pathway. The transcription factor early B‐cell factor 2 (EBF2) regulates the expression of key genes, including FGF7, within this subpopulation, thereby further strengthening FGF7–FGFR2 signalling and promoting peritoneal metastasis. These findings were validated using PDOs and corresponding in vivo models. Taken together, our findings indicate that inhibition of the FGF–FGFR signalling pathway could offer a potential therapeutic approach for gastric cancer with peritoneal metastasis.

## 2 MATERIALS AND METHODS

### 2.1 Tissue samples

Tissue samples from peritoneal metastases were collected from patients at the First Affiliated Hospital of Soochow University. These specimens served as the source material for single‐nucleus transcriptomic profiling, PDO generation and in vivo studies. Ethical approval was granted by the Institutional Ethics Committee (approval no. 2022164), and the study involving human specimens was conducted in compliance with the Declaration of Helsinki.

### 2.2 Single‐nucleus RNA sequencing analysis

snRNA‐seq analysis was performed by Beijing Novogene Technology Corporation. The bioinformatics analysis methods used are as follows.

#### 2.2.1 Cell‐type annotation

Following normalisation of the snRNA‐seq expression matrix, highly variable genes were selected for downstream dimensionality reduction and clustering. Principal component analysis (PCA) and unsupervised clustering were performed using Seurat (v4.3.0). Cell identities and subpopulations were subsequently determined according to established marker genes, with annotations supported by information from the CellMarker database and relevant published literature. The assigned cell types were further examined and manually validated based on their distributions in uniform manifold approximation and projection (UMAP) and t‐distributed stochastic neighbor embedding (t‐SNE) projections.

#### 2.2.2 Copy number variation analysis (inferCNV)

Chromosomal copy number variations (CNVs) in tumour cells were analysed using inferCNV (v1.12.1). Normal cell populations were selected as references. Gene expression was normalised using a sliding window approach to identify signals of copy number gains and losses. Heatmaps were generated to visualise genomic instability and clonal heterogeneity in tumour cells.

#### 2.2.3 Pseudotime analysis

Monocle3 (v0.3.3) was used to construct pseudotime trajectories for specific cell populations. Highly variable genes were selected, UMAP dimensionality reduction was applied, and pseudotime was calculated to reconstruct cellular developmental or state transition pathways. Pseudotime analysis was employed to infer dynamic changes in cell states and potential differentiation trajectories.

#### 2.2.4 Transcriptional regulatory network analysis (pySCENIC)

Transcriptional regulatory networks were reconstructed with pySCENIC (v0.11.2). GRNBoost2 was employed to determine co‐expression associations linking transcription factors to candidate target genes. The resulting regulatory modules were filtered against the cisTarget database so that only putative direct targets were retained. AUCell subsequently calculated the activity score of each transcription factor on a cell‐by‐cell basis. This approach enabled the identification of major transcription factors and their associated regulatory modules underlying differences in gene expression among cell states.

#### 2.2.5 Cell–cell interaction analysis

Potential communication events between cell populations were predicted with CellChat or CellPhoneDB (v4.0) using the normalised single‐nucleus RNA‐sequencing (snRNA‐seq) expression matrix together with the corresponding cell‐type labels. Ligand and receptor genes detected in at least 10% of nuclei in the relevant cell populations were included in the analysis. Predicted interactions were presented as network plots and heatmaps to depict the principal signalling pathways and patterns of communication between cell populations.

#### 2.2.6 NicheNet analysis

NicheNet v2 was used to perform intercellular communication analysis using both sender‐agnostic and sender‐focused approaches. The sender‐agnostic method considers the expression of ligands corresponding to receptors in the target cells, while the sender‐focused method further filters for ligands also expressed in the ligand‐expressing cells. The results were saved in the directories ‘sender_agnostic’ and ‘sender_focused’, respectively, for inferring potential regulatory effects of ligands on target cell states.

#### 2.2.7 Functional enrichment analysis

Gene Ontology (GO) enrichment was evaluated with the clusterProfiler package after accounting for gene‐length bias. Results with ∖(*p* < .05∖) were regarded as statistically significant. The same package was employed for Kyoto Encyclopedia of Genes and Genomes (KEGG) pathway analysis to determine the significance of marker‐gene enrichment in individual KEGG pathways. Reactome pathway enrichment was examined with the ReactomePA package. Protein–protein interaction (PPI) networks for the marker genes were generated through STRINGdb.

### 2.3 Cell lines and cell culture

Human gastric cancer cell lines MKN45 (RRID: CVCL_0434) and SNU‐1 (RRID: CVCL_0099) were employed in this study. MKN45 was sourced from Procell Life Science & Technology Corporation, while SNU‐1 was supplied by Shanghai FuHeng Biotechnology Co., Ltd. Both cell lines were routinely screened for mycoplasma and verified to be contamination‐free. Cells were propagated in RPMI 1640 or DMEM supplemented with 10% foetal bovine serum (FBS; Vitalizer) and 1% penicillin/streptomycin (HyClone). The cultures were incubated at 37°C under humidified conditions with 5% CO_2_ and 95% air.

### 2.4 Immunofluorescence

#### 2.4.1 Immunofluorescence of cells and organoids

Cells were cultured in six‐well plates (Biosorfa) or glass‐bottom dishes (Biosharp). Upon reaching approximately 60%–70% confluence, the medium was discarded, and the cells were rinsed with phosphate‐buffered saline (PBS; Vitalizer). The cells were then treated with 4% paraformaldehyde (Biosharp) for fixation, followed by membrane permeabilisation with Triton X‐100 (Sigma‐Aldrich) and blocking with 1% bovine serum albumin (BSA; Sigma‐Aldrich). The designated primary antibodies were applied, and the cells were maintained at 4°C overnight. After washing away any unbound primary antibodies, the corresponding fluorescence‐conjugated secondary antibodies (Beyotime) were applied. Cell nuclei were labelled with Hoechst 33342 (Beyotime). Fluorescence signals were examined and recorded using a confocal laser‐scanning microscope (OLYMPUS Corporation). For immunofluorescence analysis of organoids, the organoids were harvested into centrifuge tubes (Promethe), processed and placed onto slides (Citotest). Subsequent staining was carried out according to the immunofluorescence procedure detailed above, and microscopic images of the stained organoids were acquired for further analysis.

#### 2.4.2 Immunofluorescence of tissues

Paraffin‐embedded sections were processed with deparaffinisation solution (Servicebio), passed through a graded ethanol series and rehydrated in water. Frozen sections were dried at 37°C, immersed in methanol (Sinopharm Chemical Reagent Co., Ltd.) for fixation, and rinsed with PBS. Cells grown on coverslips and cell smears were fixed in 4% paraformaldehyde and treated with Triton X‐100 for membrane permeabilisation, followed by PBS rinsing. The permeabilisation step was omitted when membrane proteins were examined. Suspended cells were harvested by centrifugation and used to prepare smears, which were air‐dried before fixation and permeabilisation. When required, tissue sections underwent antigen retrieval and were rinsed with PBS after cooling naturally. Before immunostaining, the sections were incubated with BSA or a suitable serum for 30 min to block non‐specific binding and then exposed to primary antibodies at 4°C overnight. Following PBS washing, fluorescence‐labelled secondary antibodies (Servicebio) were applied, and the sections were kept at room temperature in darkness. Cell nuclei were stained with 4′,6‐diamidino‐2‐phenylindole (DAPI) (Servicebio). Autofluorescence was quenched when necessary. The sections were subsequently coverslipped with antifade mounting medium (Beyotime), and fluorescence images were visualised and acquired using an OLYMPUS fluorescence microscope (OLYMPUS Corporation). The antibodies used and their catalogue numbers are as follows: SEMA3C (Novus Biologicals, Cat# MAB1728), CD31 (Servicebio, Cat# GB11063‐1), VWF (Servicebio, Cat# GB11020), FGFR2 (Proteintech, Cat# 13042‐1‐AP), PDGFRβ (Epizyme, Cat# P012976), VEGFR2 (Epizyme, Cat# P013400), CK7 (Biodragon, Cat# BD‐PM3054), Ki67 (Servicebio, Cat# GB111499) and EBF2 (Affinity, Cat# DF13398).

### 2.5 Organoid construction and viability assay

Fresh tumour samples were promptly immersed in primary tumour tissue preservation solution (Mogengel Bio; Cat. No. MB‐0818L04). After the preservation medium was discarded, the samples were washed with Dulbecco's phosphate‐buffered saline (DPBS) containing 1% penicillin/streptomycin. Sterile scissors and forceps (Beyotime) were used to excise the necrotic and non‐parenchymal regions. The retained tumour tissue was enzymatically dissociated in primary tumour tissue digestion solution (Mogengel Bio; Cat. No. MB‐0818L05), and FBS (Vitalizer) was subsequently added to terminate digestion. After centrifugation, the collected tissue fraction was treated with erythrocyte lysis solution (Mogengel Bio; Cat. No. MB‐0818L08) and washed again with DPBS. The obtained cell suspension was embedded in matrix gel (Mogengel Bio; Cat. No. 082755) and seeded into 24‐well plates (Beyotime). The cells were cultured in complete medium for gastric cancer organoids (Mogengel Bio; Cat. No. MA‐0807T008LP) until organoid formation was observed. Organoid viability was subsequently evaluated with an organoid viability assay kit (BioGenous; Cat. No. E238004).

### 2.6 HUVEC culture and tumour‐associated endothelial cell construction

Human umbilical vein endothelial cells (HUVECs; FuHeng Biotechnology) were cultured in complete medium containing 10% FBS and 1% penicillin/streptomycin (Sangon Biotech) and maintained in a 37°C, 5% CO_2_ humidified incubator. When cells reached 80%–90% confluence, they were passaged using trypsin (Vitalizer) and seeded into appropriate culture plates according to experimental requirements. During culture, endothelial cell‐specific medium (FuHeng Biotechnology) was regularly replaced to maintain optimal cell growth for subsequent experiments. TECs were generated when HUVECs were in a healthy growth state. Supernatant from gastric cancer metastatic organoid cultures was collected and used to stimulate HUVECs. After two to three passages of stimulation, the TECs were used for downstream experiments.

### 2.7 Organoid and tumour‐associated endothelial cell co‐culture

A layer of matrix gel (Mogengel Bio, Cat: 082755) was applied to the bottom of 24‐well plates (Biosorfa). The matrix gel could be diluted with DPBS at an appropriate ratio. After gel solidification, well‐grown organoids were seeded into the plates. TECs in the logarithmic growth phase were collected, fully resuspended in organoid complete medium and added to the wells containing organoids to establish a co‐culture system. The co‐culture could be observed under a bright‐field microscope, and a relatively stable co‐culture model was formed after approximately 1 week for subsequent experiments.

### 2.8 Haematoxylin and eosin staining

Haematoxylin and eosin (H&E) staining was performed for morphological observation of cell crawls and tissue sections. For cell crawls, cells were fixed, permeabilised and washed, followed by pretreatment, sequential haematoxylin staining, differentiation and bluing, then counterstained with eosin, dehydrated with ethanol and mounted. For tissue sections, paraffin sections were deparaffinised to water, and frozen sections were allowed to return to room temperature and fixed. Sections were then pretreated, haematoxylin‐stained, differentiated, blued and eosin‐stained (Servicebio). After staining, tissue sections were sequentially dehydrated with graded ethanol, cleared and mounted with neutral resin (Solarbio). Observations and image acquisition were performed using an optical microscope.

For organoids, organoid pellets were first collected by centrifugation and fully embedded in 1.5%–3% liquid agarose (Yisheng Bio). After the agarose cooled and solidified into a block, the agarose‐embedded organoids were subjected to routine dehydration and embedding, providing samples for subsequent sectioning and H&E staining.

### 2.9 Enzyme‐linked immunosorbent assay

SEMA3C concentrations were measured using a commercial sandwich enzyme‐linked immunosorbent assay (ELISA) kit (Shanghai Aimeng Youning Biotechnology Co., Ltd., Cat# LV11678) according to the manufacturer's protocol. Briefly, samples and serially diluted standards were applied to antibody‐coated plates and subjected to sequential incubation with biotinylated detection antibody and SABC working solution, with washing steps performed between incubations. 3,3′,5,5′‐tetramethylbenzidine (TMB) was then used for colour development in the dark, and the reaction was terminated before absorbance measurement at 450 nm. SEMA3C concentrations were calculated from the standard curve, with samples outside the assay range appropriately diluted. Serum, plasma, tissue homogenates and cell culture supernatants were analysed as applicable. Blank wells containing sample diluent were included as controls. Samples were handled to minimise hemolysis and contamination and stored at 2–8°C for short‐term or −20°C/−80°C for long‐term preservation. Wash buffer was diluted 1:20 before use, and TMB components were mixed at a 1:1 ratio immediately before application.

### 2.10 Immunohistochemistry

Immunohistochemistry (IHC) staining was performed on 5‐µm‐thick paraffin‐embedded sections, including clinical specimens, mouse tumour samples and organoid samples, following previously reported methods.^19^, ^20^ Primary antibodies and their dilutions were used as follows. Stained sections were scanned using a NanoZoomer S60 (Hamamatsu Photonics) and analysed with ImageJ software. Antibodies used include FGFR2 (Proteintech, Cat# 13042‐1‐AP), CK7 (Biodragon, Cat# BD‐PM3054) and Ki67 (Servicebio, Cat# GB111499).

### 2.11 Agarose gel electrophoresis

A 1 × Tris‐acetate‐EDTA buffer (TAE) solution (Sangon Biotech) was used to prepare the agarose gel, with Gel‐Red fluorescent dye (Beyotime) incorporated into the gel before casting. The mixture was heated to ensure complete dissolution, with a final agarose concentration of 2%–3%. DNA obtained from chromatin immunoprecipitation (ChIP) experiments was amplified, mixed with appropriate loading buffer (Vazyme) and subjected to agarose gel electrophoresis. Electrophoresis conditions were 100 V, 100 mA for 50–60 min. Band position and size were visualised to verify enrichment of specific DNA fragments.

### 2.12 Luciferase reporter assay

The plasmid pcDNA3.1‐FGF7, containing the FGF7 promoter upstream of the firefly luciferase reporter gene, was constructed (Hangzhou Guannan Biotechnology Co., Ltd.). SNU‐1 cells (RRID: CVCL_0099; FuHeng Biotechnology) were transduced with either EBF2 overexpression or empty vector plasmids. When cells reached 60%–80% confluence, pcDNA3.1‐FGF7 plasmid was transfected. The following day, cells were lysed, and luciferase activity was measured using the Dual‐Luciferase Reporter Assay System (Yisheng Bio), with Renilla luciferase activity used for normalisation.

### 2.13 Chromatin immunoprecipitation

ChIP experiments were conducted with the ChIP Assay Kit (Beyotime Biotech). Cells were treated with 1% formaldehyde for 10 min to crosslink DNA and proteins, after which the reaction was quenched. Cell lysates were subsequently obtained, and the chromatin was fragmented into 200–500 bp segments using a Bioruptor sonicator. The fragmented chromatin was incubated at 4°C overnight with an anti‐EBF2 antibody (R&D Systems, Cat. No. AF7006), an anti‐H3 antibody as the positive control (Proteintech, Cat. No. 17168‐1‐AP) or agarose beads alone as the negative control. Protein A agarose beads were then added to capture the antibody‐bound chromatin complexes for 1 h. After purification, the recovered DNA was subjected to qPCR with SYBR Green PCR Master Mix (Novoprotein) to determine fold enrichment. The FGF7 primer sequences were as follows: FP: GTTCTAACATTCTGAGCACAAAGA; RP: TTGCTGACCTCATTGGAAGCA.

### 2.14 Animal models construction

Five‐ to 6‐week‐old non‐obese diabetic severe combined immunodeficient (NOD–SCID) mice weighing 16–18 g were used to generate PDOX models. Mature organoids were harvested, pelleted by centrifugation, rinsed with DPBS, mixed with concentrated matrix gel and subsequently inoculated into the subcutaneous tissue. For the generation of PDX models, freshly obtained tumour specimens were cut into approximately 2 × 2 × 2 mm^3^ fragments. These fragments were transplanted into the designated sites of recipient mice of comparable age and body weight using a puncture needle. Tumour volume was estimated according to the equation *V* = .5 × *L* × *W* ^2^, where *L* and *W* represent tumour length and width, respectively. Once tumour growth was confirmed, the animals were allocated at random to the designated experimental groups, with five mice included in each group. Each mouse was regarded as an independent experimental unit for statistical analysis. Tumour dimensions were recorded at prespecified intervals following a consistent measurement procedure to reduce potential measurement bias. All animal procedures, including euthanasia, were conducted in accordance with the animal welfare requirements of Soochow University and applicable AAALAC standards. The experimental protocol was reviewed and approved by the Institutional Animal Care and Use Committee (IACUC) of Soochow University (approval no. 202406A0602).

### 2.15 Kaplan–Meier survival analysis

The prognostic relevance of SEMA3C transcript abundance for post‐progression survival (PPS) in patients with gastric cancer was investigated through the Kaplan–Meier Plotter platform (https://kmplot.com/analysis/). SEMA3C expression was represented by the Affymetrix probe 203788_s_at. The optimal cutoff automatically generated by the platform from the available percentile thresholds was used to stratify patients into higher‐ and lower expression cohorts. PPS distributions were estimated with the Kaplan–Meier method, and the difference between the two cohorts was examined using the log‐rank test.

### 2.16 GEPIA analysis and correlation analysis

The expression profile of SEMA3C across different pathological stages of gastric cancer was examined through the Gene Expression Profiling Interactive Analysis (GEPIA) web server using data from The Cancer Genome Atlas stomach adenocarcinoma cohort (TCGA‐STAD). SEMA3C expression values were transformed as log2(TPM + 1) and compared among pathological stages I–IV. Differences across stages were evaluated by one‐way analysis of variance. GEPIA was also employed to investigate the relationship between EBF2 and FGF7 transcript abundance. This assessment used the integrated STAD expression cohort, comprising TCGA‐STAD tumour specimens, TCGA‐STAD adjacent normal specimens and normal gastric tissues from the Genotype‐Tissue Expression (GTEx) project. The association between EBF2 and FGF7 expression was quantified using Spearman's rank correlation coefficient.

### 2.17 Transcription factor binding site prediction

Bioinformatic analyses were performed in Python (Python 3.8) using the Biopython library. The FGF7 promoter sequence was obtained from NCBI, and potential transcription factor binding sites were predicted using motif sequence information. Additionally, transcription factor position weight matrices (PWMs) were obtained from the HOCOMOCO database (https://hocomoco14.autosome.org/motif/COE2.H14CORE.0.P.B#downloadsInfo) for further analysis. The sequences of Ebf2 target genes in mouse were obtained from the website (https://jaspar.elixir.no/search?q=EBF2&collection=CORE&tax_group=vertebrates&tax_id=all&type=all&class=all&family=all&version=all). AlphaFold3 (https://alphafoldserver.com/) was used to generate an exploratory structural model of EBF2 in complex with the candidate FGF7 promoter sequence identified by motif‐based analysis. The amino acid sequence of EBF2 was retrieved from UniProt (https://www.uniprot.org/uniprotkb/Q9HAK2/entry). The results were analysed and visualised, and the binding site sequence information was extracted using PyMOL version 3.0.3.

### 2.18 Statistical analysis

Data analysis was conducted in GraphPad Prism (version 10). Continuous data are presented as the mean with standard deviation. Student's *t*‐test was applied when two groups were compared, while one‐way ANOVA was used for comparisons involving three or more groups. Survival distributions were estimated using the Kaplan–Meier method, and between‐group differences were examined with the log‐rank test. All tests were two‐tailed, and results with *p* values below .05 were considered statistically significant.

### 2.19 Data and code availability statement

The data generated and analysed in this study, including the raw snRNA‐seq data, pySCENIC transcription factor regulatory network analysis results, CellChat cell–cell communication analysis results and relevant data analysis scripts, are available from the corresponding author upon reasonable request.

### 2.20 Ethics statement

Written informed consent was obtained from all participants before sample collection. The study protocol was reviewed and approved by the Institutional Ethics Committee of the First Affiliated Hospital of Soochow University (approval No. 2022164). All animal experiments were approved by the Institutional Animal Care and Use Committee (IACUC) of Soochow University (approval no. 202406A0602). All animal procedures, including euthanasia, were conducted in accordance with the animal welfare requirements of Soochow University and applicable AAALAC standards.

## 3 RESULTS

### 3.1 Single‐nucleus profiling of SEMA3C^+^ tumour cells in peritoneal metastasis

To investigate tumour heterogeneity and the potential molecular mechanisms underlying peritoneal metastasis of gastric cancer, we performed snRNA‐seq on metastatic tissue samples obtained from six patients with GCPMs. Based on cell‐type annotation (Figure 1A,B), major cellular populations were classified as lymphocytes, myeloid cells, fibroblasts, myofibroblasts, epithelial cells and endothelial cells. Representative marker genes for each cell type are shown as violin plots (Figure 1C). The proportions of these major cell types varied across individual samples (Figure 1D).

**FIGURE 1:**
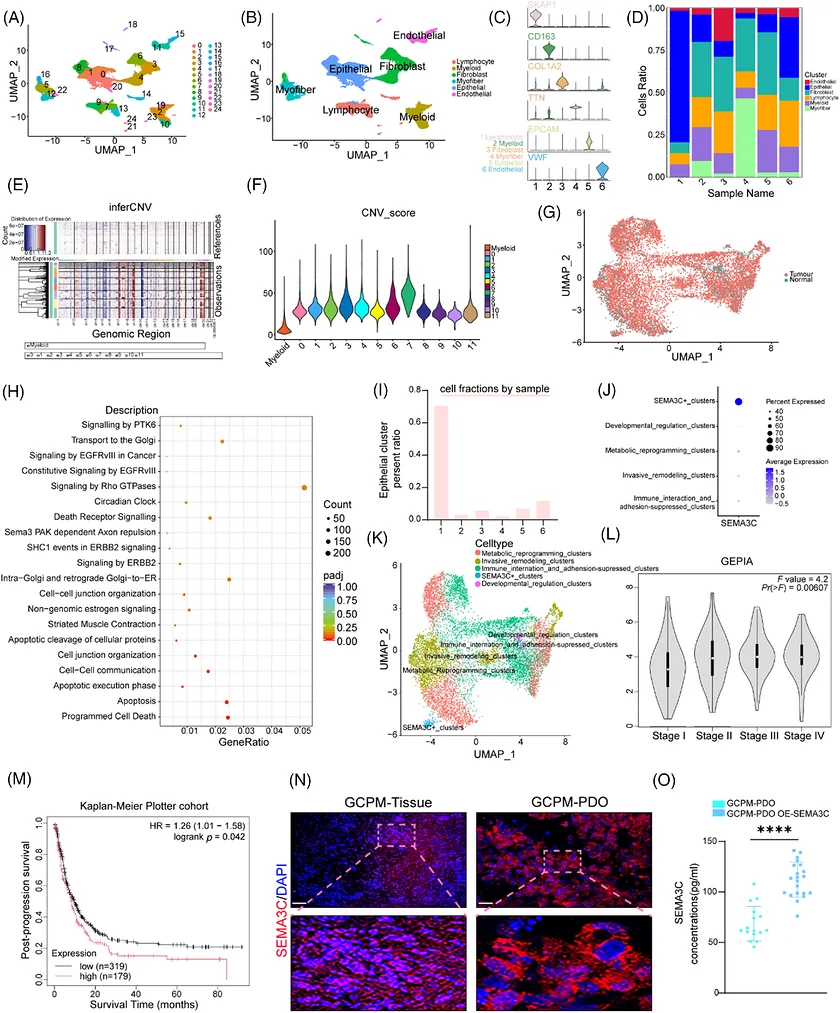
Single‐nucleus profiling of SEMA3C^+^ tumour cells in peritoneal metastasis. (A) Uniform manifold approximation and projection (UMAP) dimensionality reduction showing unsupervised clustering of all cells across the samples. (B) UMAP plot displaying annotated cell subpopulations, grouped into six major categories: lymphoid, myeloid, fibroblast, myofibroblast, epithelial and endothelial cells. (C) Violin plots of marker gene expression used for cell type annotation. (D) Bar plots showing the proportion of each major cell type across the individual samples. (E) inferCNV analysis of epithelial cells. (F) Violin plots comparing copy number variation (CNV) scores between reference myeloid cells and epithelial cells across clusters 0–11. (G) UMAP plot depicting the distribution of normal versus tumour epithelial cells after inferCNV analysis. (H) Functional enrichment analysis of epithelial cell populations. (I) Bar plots illustrating the proportion of epithelial cells across the different samples. (J) Bubble plot showing semaphorin 3C (SEMA3C) expression across annotated epithelial subpopulations. (K) UMAP visualisation of epithelial cell subpopulations after annotation. (L) SEMA3C expression levels across different pathological stages of gastric cancer based on the Gene Expression Profiling Interactive Analysis (GEPIA) platform using the TCGA‐STAD dataset. (M) Kaplan–Meier survival analysis showing the association between SEMA3C expression and post‐progression survival (PPS) in gastric cancer patients using the Kaplan–Meier Plotter database. (N) Representative immunofluorescence images showing SEMA3C expression in gastric cancer peritoneal metastases and patient‐derived organoids (scale bar: 50 µm). (O) Dot plots of SEMA3C levels measured by enzyme‐linked immunosorbent assay (ELISA) in conditioned media from different experimental groups.

Gastric cancer cells are primarily derived from epithelial cells. InferCNV analysis of epithelial cells (Figure 1E) revealed that subpopulations within metastatic lesions exhibited pronounced CNV signals, indicative of malignant transformation (Figure 1F). At a clustering resolution of .05, the vast majority of epithelial cells were annotated as tumour cells, with only a small fraction identified as normal epithelial cells (Figure 1G), further confirming the predominantly malignant nature of epithelial cells in our samples.

Functional enrichment analysis of the tumour cell population demonstrated significant associations with biological processes including cell death, intercellular communication, signalling pathways, organelle transport and axon repulsion (Figure 1H). The distribution of epithelial cells across different samples was also quantified (Figure 1I). Among these functional categories, axon repulsion—a mechanism that regulates axonal growth direction through repulsive signalling during neural development—has been implicated in cancer cell migration in certain contexts. Further subclustering analysis identified a distinct tumour cell subpopulation characterised by high expression of SEMA3C, clearly distinguishing it from other tumour cell subsets (Figure 1J). UMAP visualisation further demonstrated that this subpopulation was well segregated from other tumour clusters (Figure 1K). Gene set enrichment analysis (GSEA) of additional tumour cell subpopulations revealed distinct functional profiles, and cells were subsequently classified and annotated based on these functional characteristics (Figure S1A–D).

As a member of the semaphorin protein family, SEMA3C is known to regulate axon repulsion. Its high expression in tumour epithelial cells suggests a potential role in promoting tumour cell migration and invasion. Further analysis of public datasets provided supportive evidence for the association of SEMA3C with gastric cancer progression and patient prognosis: SEMA3C expression was elevated in advanced gastric cancer samples (Figure 1L), and higher SEMA3C expression was associated with poorer prognosis (Figure 1M).

Immunofluorescence analysis demonstrated high SEMA3C expression in tumour cells from GCPMs as well as in corresponding PDOs (Figure 1N). ELISA analysis of organoid culture supernatants demonstrated that SEMA3C was present in the culture supernatant, and SEMA3C overexpression further increased its concentration (Figure 1O).

### 3.2 SEMA3C^+^ tumour cells exhibit stem‐like potential and endothelial interactions

To further explore the developmental and differentiation trajectories of tumour cell subpopulations, we performed pseudotime trajectory analysis. The SEMA3C^+^ subpopulation was found to reside in a less differentiated state and exhibited the highest differentiation potential, representing the origin of the overall differentiation trajectory. This subpopulation therefore serves as a key focus for investigating tumour development, differentiation and cell fate regulation (Figure 2A–C). These findings suggest that high SEMA3C expression in this subpopulation may be associated with the maintenance of tumour cell stemness. Based on this, we further analysed the correlation between SEMA3C expression and the TPM levels of stemness‐related marker genes in GCPM tumour cells. The results showed that SEMA3C was positively correlated with multiple stemness‐associated marker genes, suggesting that SEMA3C may play an important role in maintaining the stem‐like state of tumour cells (Figure S2A–D). A heatmap highlighted genes that were highly expressed along trajectory‐dependent branches throughout the differentiation process (Figure S2E).

**FIGURE 2:**
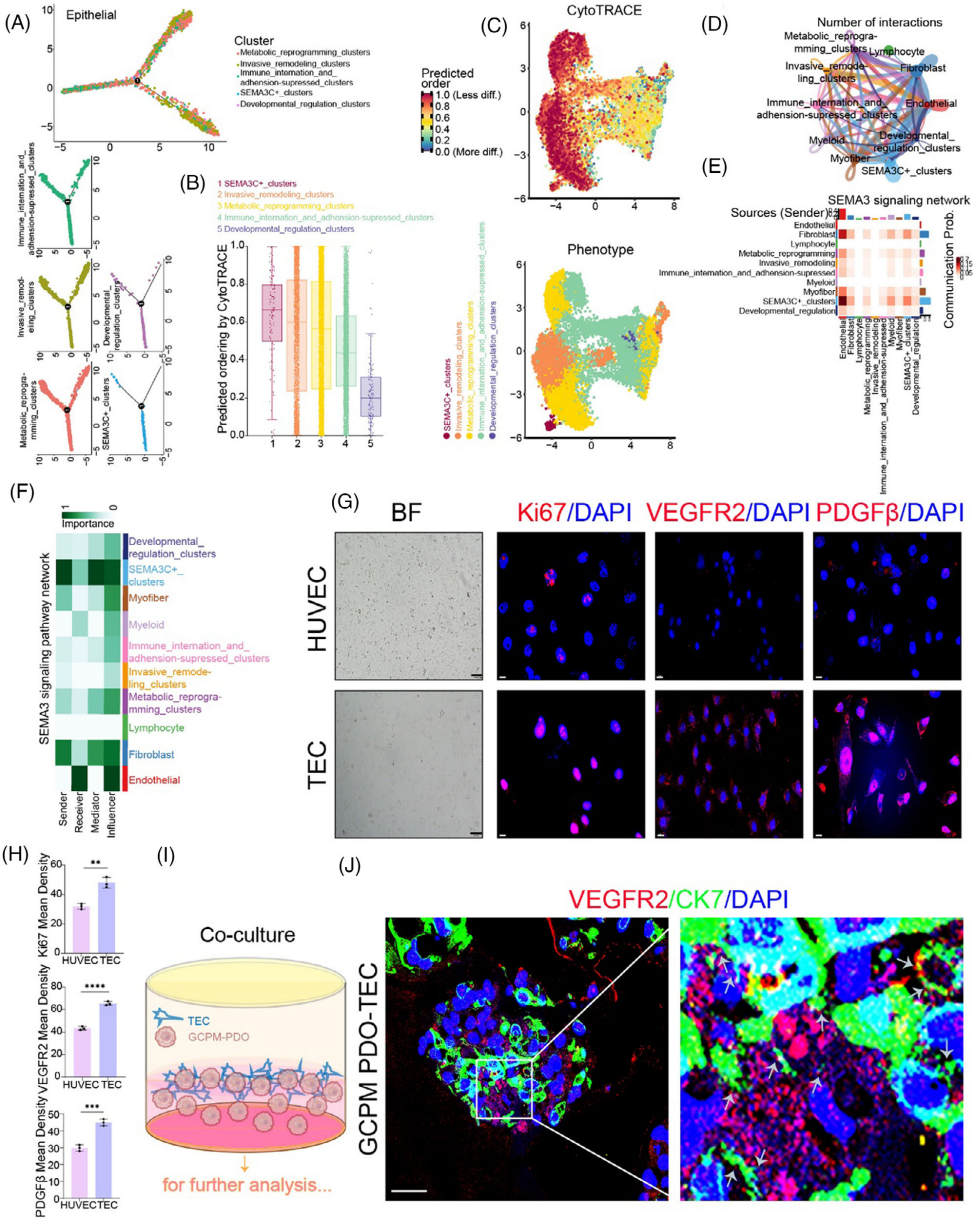
SEMA3C^+^ tumour cells exhibit stem‐like potential and endothelial interactions. (A) Pseudotime trajectory analysis of epithelial cell subpopulations, showing differentiation paths. (B) Box plots comparing differentiation states among epithelial cell subpopulations. (C) CytoTRACE analysis depicting differentiation status across epithelial subpopulations. (D) Circos‐style plot illustrating interaction strength between epithelial subpopulations and other major annotated cell types; arc thickness represents signal intensity. (E) Heatmap showing activation levels of the SEMA3 signalling pathway across cell subpopulations. (F) Cell–cell interaction heatmap displaying intercellular signalling activity. (G) Bright‐field and immunofluorescence images showing morphology and marker expression of human umbilical vein endothelial cells (HUVECs) and tumour‐associated endothelial cells (TECs); bright‐field scale bar: 100 µm, fluorescence scale bar: 20 µm. (H) Bar plots quantifying fluorescence intensity between HUVECs and TECs. (I) Schematic diagram of the gastric cancer peritoneal metastasis (GCPM) PDO–TEC co‐culture system. (J) Immunofluorescence images of co‐cultured organoids and TECs, showing relevant marker expression (scale bar: 30 µm). The panels on the right represent enlarged views of the boxed areas.

Cell–cell interaction analysis between tumour subpopulations and other major cell types revealed that the SEMA3C^+^ tumour cells interacted most extensively with endothelial cells (Figure 2D). Notably, SEMA3C‐related signalling pathways were significantly activated in these tumour–endothelial interactions (Figure 2E,F). To validate the molecular mechanisms underlying these interactions, we stimulated HUVECs with organoid culture supernatant to generate TECs. Prior to co‐culture, we confirmed endothelial identity by verifying the expression of canonical markers, including high levels of CD31 and VWF (Figure S2F).

Bright‐field imaging revealed that TECs formed looser intercellular connections compared to controls (Figure 2G). Immunofluorescence analysis showed that TECs exhibited elevated expression of proliferation and angiogenesis markers, including Ki67, VEGFR2 and PDGFβ (Figure 2G,H). Subsequently, a co‐culture model of tumour organoids and TECs was established (Figure 2I). Immunofluorescence confirmed that tumour organoids strongly expressed CK7, whereas TECs were dispersed within the organoids and displayed high VEGFR2 expression (Figure 2J), demonstrating close cellular crosstalk between tumour and endothelial compartments.

### 3.3 FGF–FGFR mediates endothelial–tumour crosstalk

Subpopulation annotation of endothelial cells revealed distinct subsets with specialised transcriptional programs (Figure 3A,B). Pseudotime trajectory analysis indicated a differentiation progression from venous endothelial cells towards connective endothelial cells (Figure 3C–E). A heatmap depicted genes highly expressed along trajectory‐dependent branches during endothelial differentiation (Figure 3F). Cell–cell interaction analysis further confirmed substantial interactions between endothelial subpopulations and SEMA3C^+^ tumour cells (Figure 3G). Among the signalling pathways enriched in endothelial cells, the fibroblast growth factor (FGF) pathway was prominently activated and mediated communication with the SEMA3C^+^ tumour subpopulation (Figure 3H,I). NicheNet analysis suggested that FGF–FGFR signalling was significantly activated in tumour cells, highlighting its potential role in tumour–endothelial crosstalk (Figure 3J). Although different analyses identified partially overlapping FGFR family members, FGFR2 was consistently detected. CellChat analysis further showed that, within SEMA3C^+^ tumour cells, FGFR2 displayed a broader expression distribution across multiple subclusters, suggesting that FGFR2 may play a more prominent role in FGF‐related intercellular communication (Figure 3K).

**FIGURE 3:**
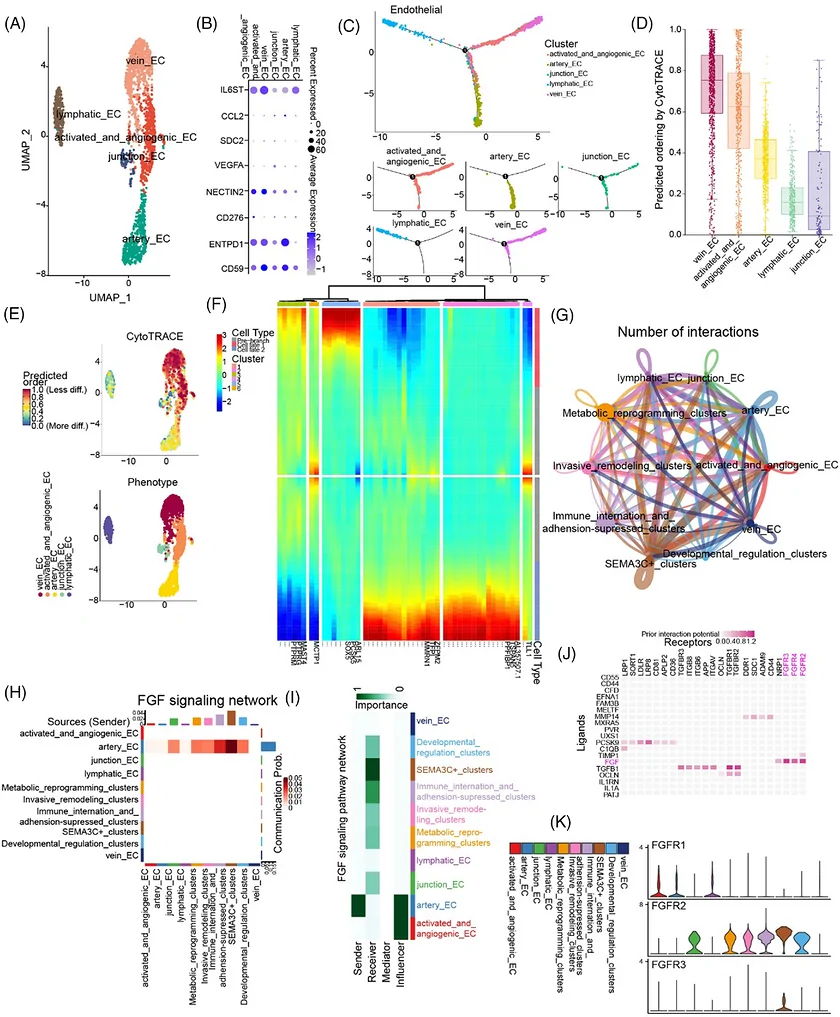
FGF–FGFR‐mediated endothelial–tumour crosstalk. (A) Uniform manifold approximation and projection (UMAP) plots showing annotation of endothelial cell subpopulations. (B) Bubble plots displaying marker genes used for endothelial subpopulation annotation. (C) Pseudotime trajectory analysis of endothelial cells, showing differentiation progression. (D) Box plots comparing differentiation states among endothelial subpopulations. (E) CytoTRACE analysis depicting differentiation status across endothelial subpopulations. (F) Heatmap of branch‐dependent highly expressed genes along the endothelial pseudotime trajectory. (G) Circos‐style plot illustrating interaction strength between endothelial subpopulations and other major annotated cell types; arc thickness represents signal intensity. (H) Heatmap showing activation levels of the fibroblast growth factor (FGF)signalling pathway across endothelial subpopulations. (I) Cell–cell interaction heatmap displaying intercellular signalling activity. (J) NicheNet analysis showing ligand–receptor distribution between tumour cells and other cell types. (K) Expression distribution of FGFR1, FGFR2 and FGFR3 across different cell subclusters.

### 3.4 EBF2‐regulated tumour cell subpopulation mediates SEMA3C effects

To further validate the functional role of the SEMA3C^+^ tumour cell subpopulation, we first assessed the viability of tumour organoids following treatment with varying drug concentrations for 48 h. Pemigatinib at 50 nM markedly inhibited organoid growth, indicating strong cytotoxicity at this concentration. Consequently, a lower dose of 10 nM was selected for subsequent experiments (Figure S3A). Using the organoid–TEC co‐culture model, we observed that SEMA3C overexpression markedly enhanced tumour–endothelial interactions, resulting in increased organoid size and greater endothelial cell contacts. Treatment with the pan‐FGFR inhibitor pemigatinib significantly attenuated these effects, reducing both organoid diameters and tumour–endothelial interactions (Figure 4A). Immunofluorescence analysis further showed that, along with the enhanced tumour–endothelial interactions, CK7 expression in tumour organoids and VEGFR2 expression in endothelial cells were both increased, whereas pemigatinib partially reversed these changes (Figure 4B).

**FIGURE 4:**
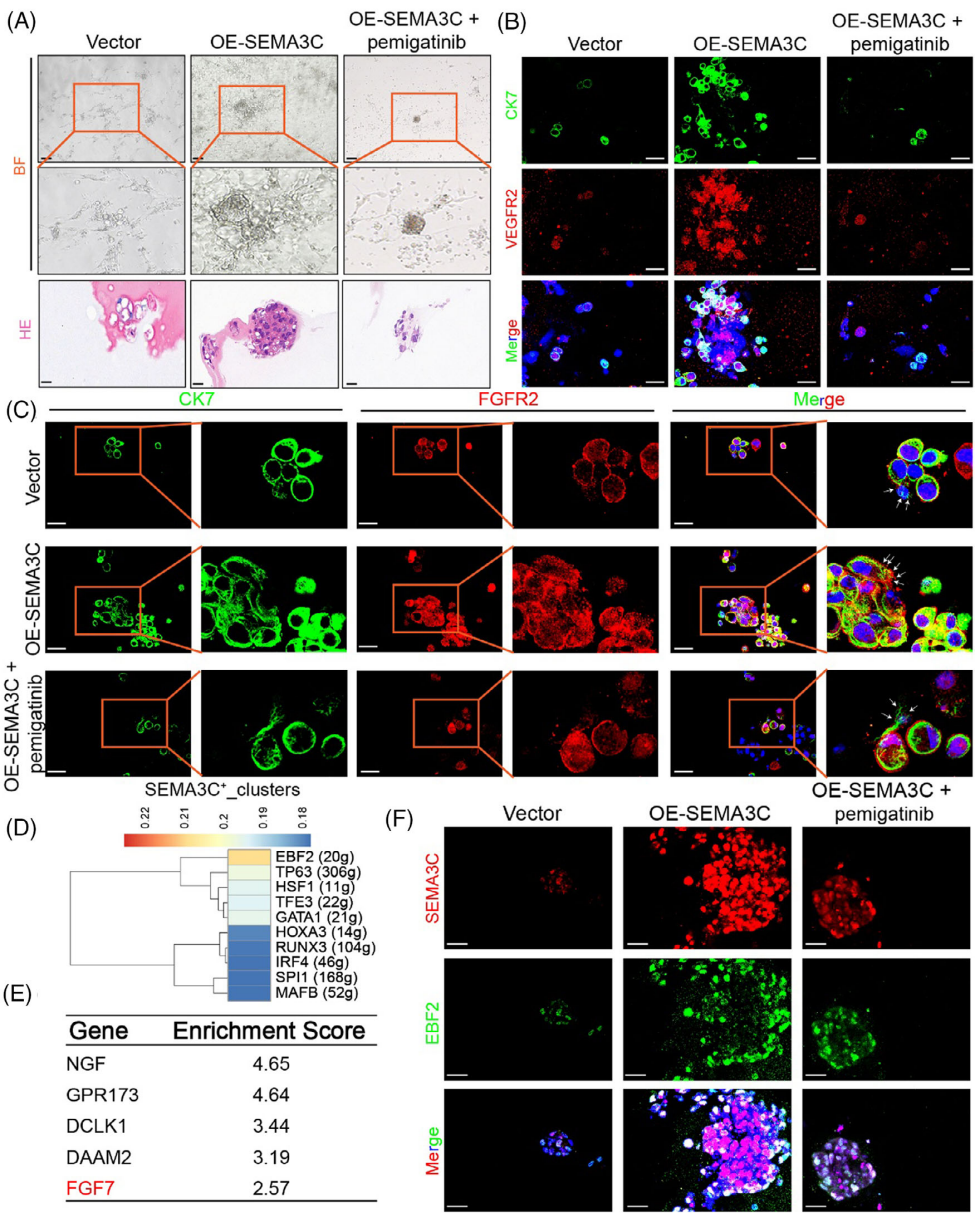
EBF2‐regulated tumour cell subpopulation mediates semaphorin 3C (SEMA3C) effects. (A) Bright‐field and haematoxylin and eosin (H&E) images showing interactions between tumour organoids and tumour‐associated endothelial cells (TECs) across different experimental groups (bright‐field scale bar: 100 µm; H&E scale bar: 20 µm). (B) Immunofluorescence images depicting the expression of relevant markers and the extent of organoid–endothelial cell interactions after co‐culture in different groups (scale bar: 30 µm). (C) Immunofluorescence images showing organoid–TEC interactions and marker expression across experimental conditions (scale bar: 30 µm). (D) pySCENIC analysis illustrating transcription factor activity within the SEMA3C^+^ tumour cell subpopulation. (E) Tripartite plot displaying predicted target genes regulated by EBF2 in the SEMA3C^+^ subpopulation. (F) Immunofluorescence images showing co‐staining of tumour cell subpopulation marker genes and predicted transcription factors under different experimental conditions (scale bar: 30 µm).

Further evaluation using CK7 as a marker of tumour organoids and FGFR2 as a readout associated with organoid–TEC interactions revealed that SEMA3C overexpression markedly enhanced tumour–endothelial crosstalk, accompanied by increased FGFR2 signal intensity. Pemigatinib treatment significantly reduced both the extent of these interactions and the fluorescence signal intensity (Figure 4C).

To elucidate the molecular mechanisms underlying SEMA3C‐mediated effects, pySCENIC analysis was performed, identifying EBF2 as the key transcription factor regulating this tumour cell subpopulation (Figure 4D). Target genes, including NGF, GPR173, DCLK1, DAAM2 and FGF7, exhibited high regulon activity scores, suggesting that they are critical downstream effectors of EBF2 (Figure 4E). Immunofluorescence further showed that, under conditions of SEMA3C overexpression accompanied by enhanced tumour–endothelial interactions, EBF2 expression was increased, whereas pemigatinib treatment reduced its expression (Figure 4F), suggesting that EBF2‐associated regulatory programs may be enhanced in this specialised tumour cell subpopulation.

### 3.5 SEMA3C drives tumour expansion via FGFR2‐mediated endothelial interaction

To further evaluate the functional role of SEMA3C in tumour progression, we established PDOX models by implanting mixtures of tumour organoids and TECs into NOD–SCID mice to generate F0 tumours. After tumour establishment, the tumours were harvested and passaged into new immunodeficient mice to generate F1 tumours. The F1 mice were then assigned to three groups: a control PDOX group derived from control organoids, a SEMA3C‐overexpression PDOX group derived from SEMA3C‐overexpressing organoids, and a SEMA3C‐overexpression PDOX group treated with the pan‐FGFR inhibitor pemigatinib (.3 mg/kg, every other day; Figure 5A).

**FIGURE 5:**
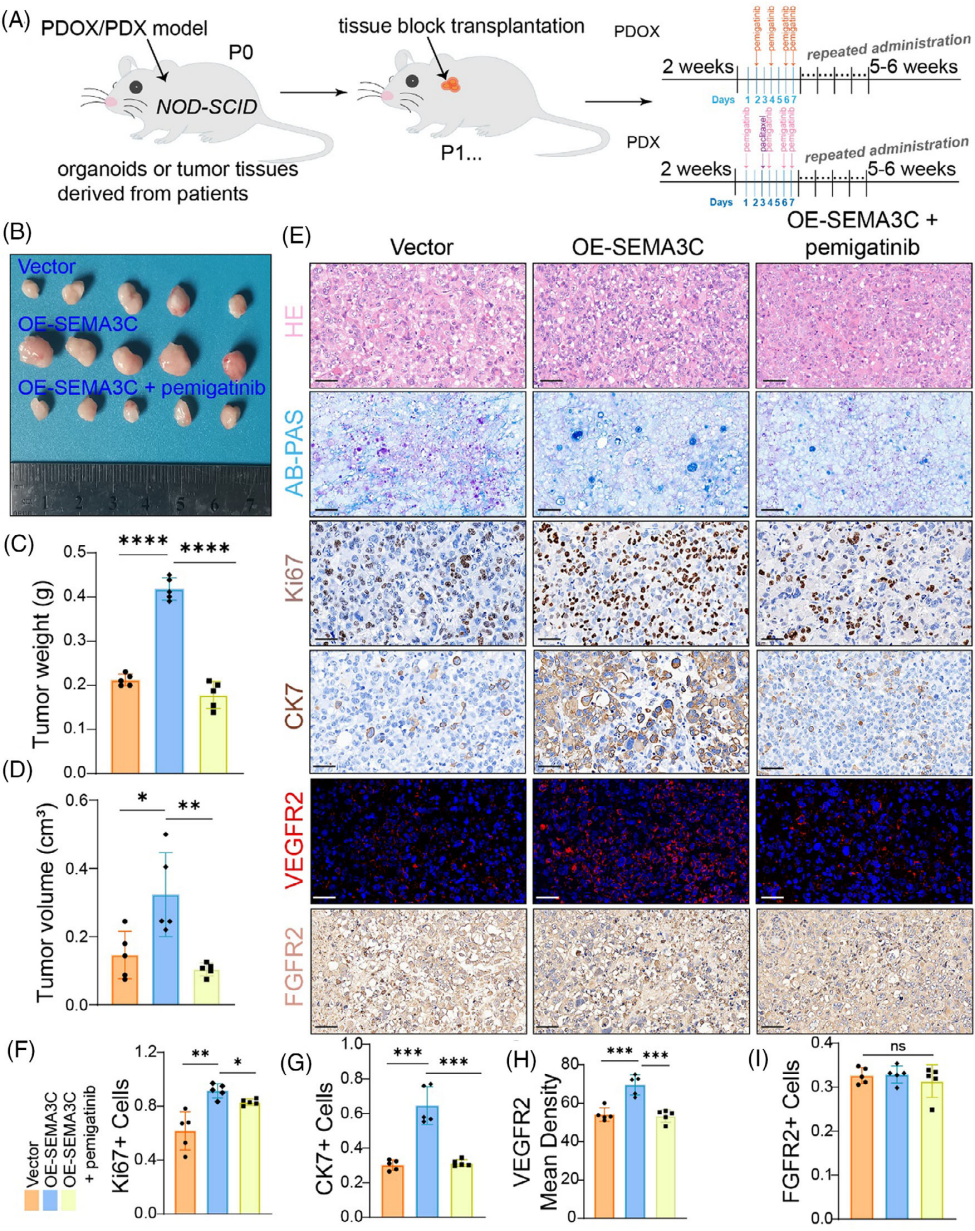
Semaphorin 3C (SEMA3C) drives tumour expansion via FGFR2‐mediated endothelial interaction. (A) Schematic diagram of the patient‐derived organoids‐based xenograft (PDOX) model construction and drug administration protocol. (B) Representative gross images of tumours from PDOX models in different experimental groups (*n* = 5 mice per group). (C) Bar graph showing tumour weights across different groups. (D) Bar graph showing tumour volumes across different groups. (E) Immunofluorescence images showing the expression of relevant markers in tumour samples from different groups (scale bar: 50 µm). (F) Quantification of Ki67‐positive cells in tumour samples across experimental groups. (G) Quantification of CK7‐positive cells in tumour samples across experimental groups. (H) Quantification of VEGFR2 fluorescence intensity in tumour samples across groups. (I) Quantification of FGFR2‐positive cells in tumour samples across groups.

After 3 weeks of treatment, tumour measurements revealed that tumours in the SEMA3C‐overexpression group were significantly larger in both size and volume compared to the control and inhibitor‐treated groups (Figure 5B–D). These results indicate that FGFR2 signalling is activated during tumour–endothelial interactions, promoting tumour cell proliferation, invasion and enhanced crosstalk with endothelial cells, thereby facilitating tumour expansion and metastasis.

Tumour tissue samples from each group were analysed for relevant molecular markers (Figure 5E). The SEMA3C‐overexpression PDOX group exhibited markedly higher proliferative activity, as evidenced by a significant increase in Ki67‐positive cells compared to control and inhibitor‐treated groups (Figure 5F). CK7‐positive tumour cells were also more abundant in the SEMA3C‐overexpression group, whereas FGFR inhibition reduced tumour cell numbers (Figure 5G). Immunofluorescence analysis of VEGFR2 demonstrated that high SEMA3C expression enhanced endothelial–tumour interactions, while inhibitor treatment decreased both the number of activated endothelial cells and fluorescence intensity (Figure 5H).

Furthermore, FGFR2 IHC revealed that FGFR2 protein was stably expressed in both tumour organoids and endothelial cells. High SEMA3C levels promoted organoid–endothelial interactions; however, FGFR inhibition did not alter FGFR2 protein expression, indicating that the inhibitor specifically targets downstream signalling activity rather than protein abundance (Figure 5I).

### 3.6 EBF2‐mediated activation of FGF7 in gastric cancer

To further investigate the regulatory role of EBF2 on its downstream target gene FGF7, we first predicted EBF2 motif characteristics using public databases. The EBF2 gene exhibits high homology between human and mouse, suggesting conserved motif patterns. Based on the sequence features of potential target genes, we identified putative EBF2 binding sites within the FGF7 promoter, with larger stars indicating higher binding likelihood (Figure 6A). Using sequence files from public resources, potential EBF2 binding sites in the FGF7 promoter were marked with red vertical lines, supporting the hypothesis that FGF7 may be a direct downstream target of EBF2 (Figure 6B). We next used AlphaFold3 to generate an exploratory in silico structural model of EBF2 in complex with a candidate FGF7 promoter sequence. The predicted model visualised a potential protein–DNA interaction and suggested putative EBF2 residues that may participate in DNA recognition (Figure 6C). However, this computational model does not constitute experimental evidence of direct EBF2 binding to the FGF7 promoter. Public transcriptomic database analysis further showed a significant positive correlation between EBF2 and FGF7 transcript levels (TPM) (Figure 6D), supporting their potential association. To experimentally validate this regulatory relationship, we constructed a luciferase reporter plasmid containing the FGF7 promoter and co‐transfected it with either an EBF2‐overexpressing plasmid or control plasmid into SNU‐1 gastric cancer cells. EBF2 overexpression significantly increased luciferase activity compared with the control, indicating that EBF2 directly promotes FGF7 transcription (Figure 6E). Chromatin immunoprecipitation followed by qPCR (ChIP‐qPCR) targeting the FGF7 promoter demonstrated markedly higher enrichment in the EBF2 antibody group relative to the beads‐only control (Figure 6F), which was further confirmed by agarose gel electrophoresis (Figure 6G–J).

**FIGURE 6:**
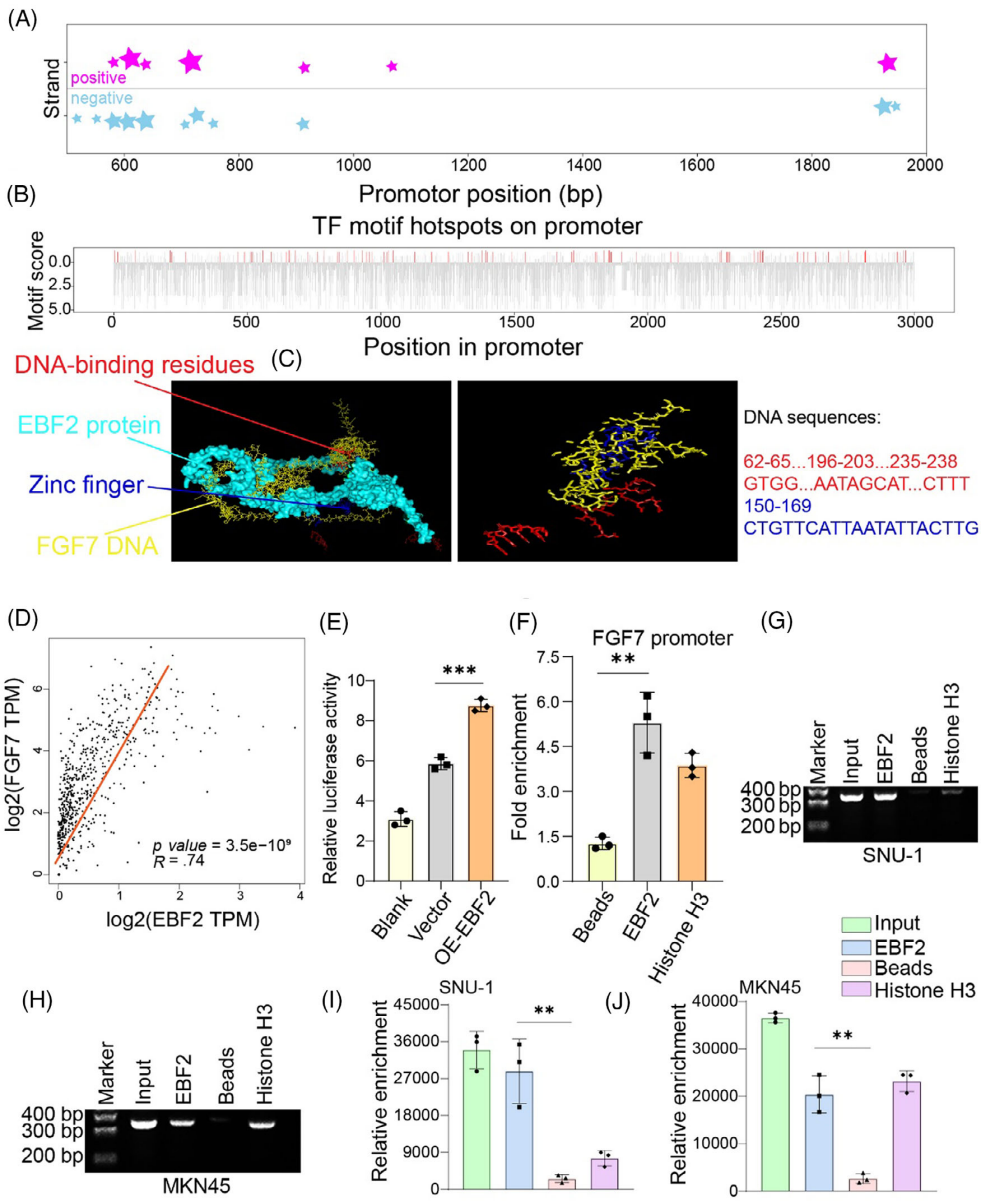
Early B‐cell factor 2 (EBF2)‐mediated activation of FGF7 in gastric cancer. (A) Predicted EBF2 binding positions on the FGF7 promoter; red indicates the sense strand, blue indicates the antisense strand and star size represents binding likelihood. (B) Heatmap showing predicted EBF2 binding sites within the FGF7 promoter (upstream 3000 bp); red vertical lines indicate potential binding sites. (C) In silico structural model of a potential interaction between EBF2 and a candidate FGF7 promoter sequence, predicted using AlphaFold3 and visualised with PyMOL. (D) Correlation analysis between EBF2 and FGF7 expression levels using the Gene Expression Profiling Interactive Analysis (GEPIA) platform based on integrated STAD datasets, including TCGA‐STAD and GTEx stomach datasets. (E) Relative luciferase activity measured in different experimental groups, indicating transcriptional regulation by EBF2. (F) Chromatin immunoprecipitation (ChIP)‐qPCR analysis showing enrichment of EBF2 binding at the FGF7 promoter in different groups. (G, H) Agarose gel electrophoresis showing FGF7 promoter fragment enrichment following ChIP in SNU‐1 (G) and MKN45 (H) cells across experimental groups. (I, J) Quantitative analysis of agarose gel electrophoresis results for SNU‐1 (I) and MKN45 (J) cells.

### 3.7 Therapeutic targeting of the EBF2–FGF7–FGFR2 signalling axis

To assess the functional relevance of the EBF2–FGF7–FGFR2 axis in vivo, we employed a PDX model. Tumour cells with high SEMA3C expression were confirmed to express EBF2 and FGF7, activating FGFR2 signalling and promoting tumour–endothelial interactions, thereby facilitating peritoneal metastasis (Figure 5A). PDX mice were treated with PBS, paclitaxel (5 mg/kg), pemigatinib (.3 mg/kg) or a combination of paclitaxel and pemigatinib. After 3 weeks, tumour size and volume measurements showed that both paclitaxel and pemigatinib significantly inhibited tumour growth, with the combination therapy exhibiting the most pronounced effect (Figure 7A,B). Analysis of tumour tissue revealed that Ki67‐positive cell numbers were markedly reduced in the paclitaxel and pemigatinib groups, with the combination group showing the greatest suppression of proliferation (Figures 7C and S3B). Immunofluorescence analysis indicated that FGFR2 protein expression remained largely unchanged across treatment groups (Figures 7C and S3C), and EBF2 protein levels were similarly unaffected (Figures 7C and S3D), suggesting that the therapeutic effects primarily involve inhibition of signalling activity rather than changes in protein abundance.

**FIGURE 7:**
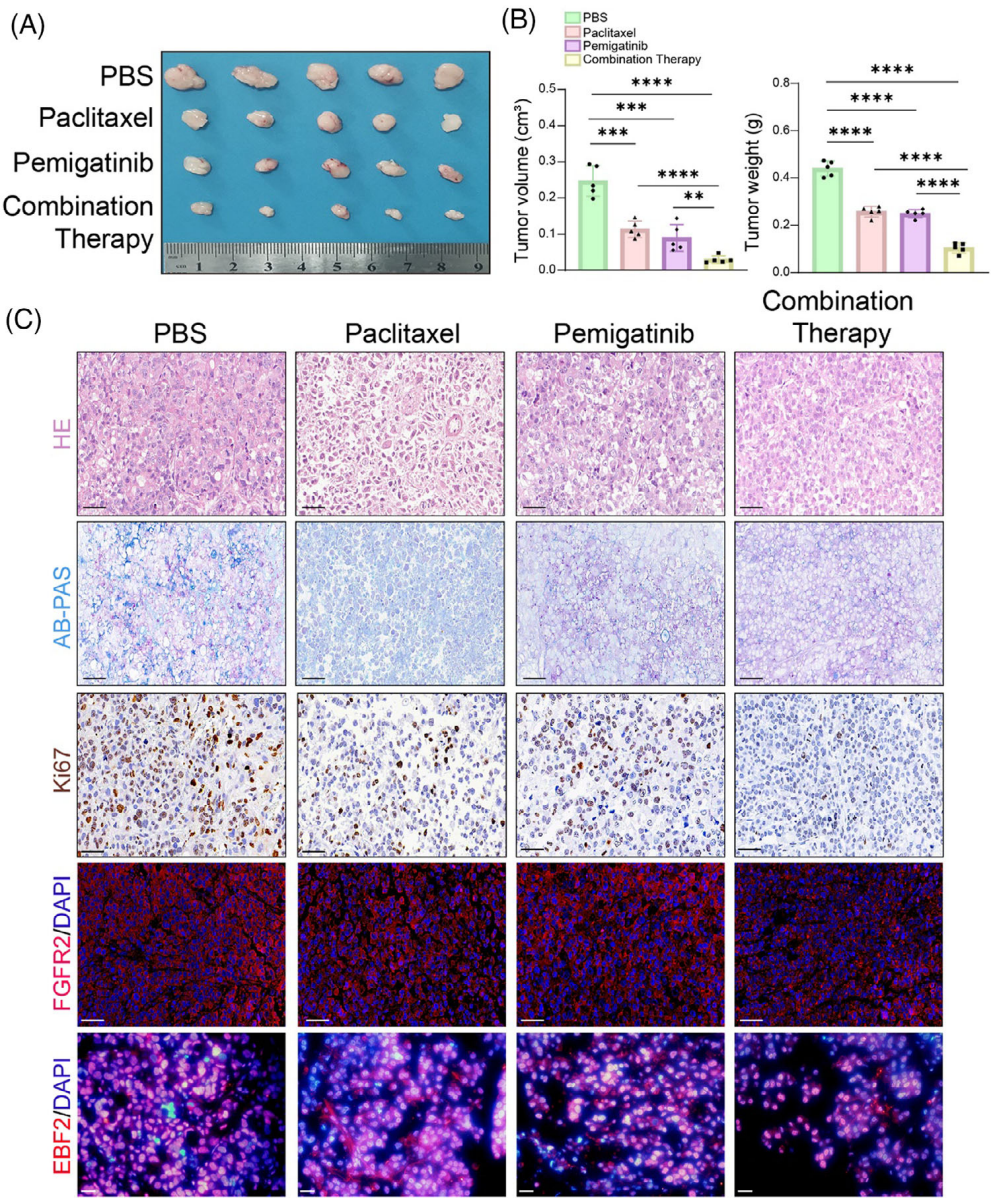
Therapeutic targeting of the EBF2–FGF7–FGFR2 signalling axis. (A) Representative gross images showing tumour sizes in patient‐derived xenograft (PDX) models across different experimental groups (*n* = 5 mice per group). (B) Bar graphs depicting tumour weights and volumes in different groups. (C) Immunofluorescence images showing the expression of relevant markers in tumour samples from different groups (scale bar: 50 µm).

## 4 DISCUSSION

This study uncovers a novel neuro–tumour mechanism underlying peritoneal metastasis of gastric cancer. Single‐nucleus transcriptomic analysis identified a subpopulation of poorly differentiated, highly stem‐like tumour cells with elevated SEMA3C expression, exhibiting pronounced metastatic potential. This subpopulation remodels the peritoneal microenvironment through axon‐inhibitory signalling, highlighting the critical role of tumour–neural interactions in shaping the metastatic niche. Mechanistically, the transcription factor EBF2 regulates FGF7 expression in this subpopulation, activating FGFR2 signalling in TECs. This axis enhances tumour–endothelial crosstalk, promotes vascular remodelling and drives tumour proliferation and invasiveness. Clinically, FGFR inhibitors, alone or in combination with chemotherapy, may represent potential therapeutic strategies for advanced GCPM. Overall, these findings demonstrate that SEMA3C^+^ tumour cells drive peritoneal metastasis via the EBF2–FGF7–FGFR2 signalling axis, providing novel experimental evidence for understanding tumour microenvironment remodelling and developing precise therapeutic interventions (Figure 8).

**FIGURE 8:**
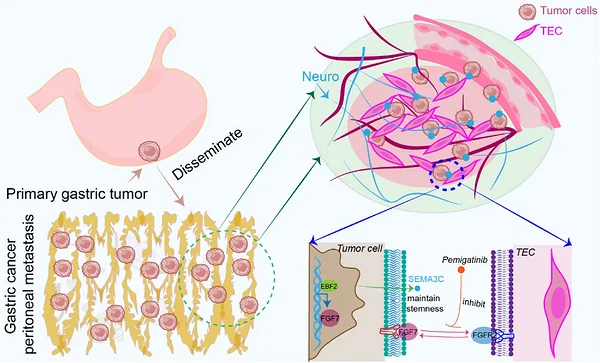
Schematic illustration summarising the key findings from single‐nucleus sequencing of gastric cancer peritoneal metastases. A tumour subpopulation with high semaphorin 3C (SEMA3C) expression was identified, which exhibits extensive interactions with tumour‐associated endothelial cells (TECs). TECs display high VEGFR2 expression, and transcriptional regulation by EBF2 in the tumour subpopulation leads to upregulation of FGF7. Tumour cells further interact with endothelial cells via the FGF7–FGFR2 signalling axis, promoting vascular remodelling, tumour proliferation and metastatic potential.

The regulation of gastric cancer progression by the nervous system has recently emerged as a significant area of interest. Studies indicate that gastric cancer cells can establish functional interactions with peripheral CGRP^+^ sensory neurons, thereby promoting tumour growth, vascular remodelling and metastasis. NGF‐dependent mechanisms drive the expansion of CGRP^+^ neurons, while sensory neuron activation enhances cancer cell proliferation and migration via calcium signalling. Inhibition of CGRP signalling or suppression of sensory neurons significantly slows tumour progression. These findings suggest that the nervous system within the gastric cancer microenvironment is not merely a passive responder but actively regulates tumour progression and metastasis.^21^ Tumour–neural interactions occur through multiple mechanisms, including direct electrochemical signalling between neurons and cancer cells, as well as paracrine signalling that influences tumour growth while being reciprocally modulated by tumour cells. Circulating neurotransmitters and neuropeptides can act on distant tumours and neural tissues, and tripartite interactions among neurons, tumour cells and immune cells modulate tumour immunity and the inflammatory microenvironment. Tumour cells may also exploit neuron‐related signals for autocrine regulation. Collectively, these interaction patterns indicate that the nervous system not only plays a critical role in tumour growth and invasion but also profoundly shapes the tumour microenvironment, offering new perspectives for understanding neuro–tumour regulatory mechanisms. Recent studies using single‐nucleus sequencing and spatial omics have further revealed key neural–immune interactions, providing a comprehensive view of tumour microenvironments at different levels of neural infiltration and establishing a theoretical foundation for targeted interventions against neural invasion in tumours.^22^

Several canonical neural genes, including NGF, BDNF, neuron‐specific proteins (Nestin, NeuN), synaptic proteins (e.g., Synaptophysin) and neurotransmitter receptor genes (e.g., NTRK family, GABR, GRIN), are aberrantly expressed in various cancers and contribute to tumourigenesis through multiple mechanisms. These genes can activate downstream signalling pathways such as MAPK/ERK, PI3K/AKT and PLCγ, directly promoting tumour cell proliferation, survival and anti‐apoptotic capacity. NGF serves as a key mediator in the tumour–brain–immune axis, enhancing tumour innervation and activating sensory–sympathetic pathways to indirectly suppress antitumour immunity.^23^ Neural genes also participate in remodelling the tumour microenvironment, reinforcing tumour–neural interactions, promoting invasion and metastasis, and regulating immune cell infiltration and angiogenesis.^24^ Markers of neural stemness, such as Nestin, are often activated in solid tumours and are associated with cancer stem cell‐like features, contributing to therapy resistance and tumour recurrence.^25^ Aberrant expression of neurotransmitter receptors and synaptic proteins can mimic neural signalling networks, facilitating intercellular communication and tumour dissemination. For example, overexpression of N‐methyl‐D‐aspartic acid (NMDA) receptors in gliomas regulates migration and proliferation via calcium signalling,^26^ while GABA receptor expression in tumours may influence tumour progression through metabolic and immune microenvironment modulation.^27^

Semaphorins, a highly conserved family of intercellular signalling molecules, exert complex, multidimensional regulatory roles in tumourigenesis and cancer progression. They influence tumour cell proliferation, migration, invasion, angiogenesis and remodelling of the immune microenvironment, with effects that are highly context‐dependent. The biological outcomes of semaphorin signalling are determined by tumour type, microenvironmental context and receptor composition, displaying both tumour‐suppressive and tumour‐promoting functions. This duality underscores the complexity of tumour microenvironment regulation and highlights semaphorins as potential targets for precision therapy.^28^, ^29^, ^30^, ^31^ Among the semaphorin family, SEMA3C—a member of the class 3 subclass—guides cell positioning during embryonic development, particularly in neural and cardiac tissues, and may facilitate tumour cell dissemination. Within the tumour microenvironment, SEMA3C expression and its cleavage products support tumour cell survival and motility, thereby regulating tumour growth and metastatic potential.^32^ Although some studies have suggested that SEMA3C can exert anti‐angiogenic effects, the prevailing evidence indicates that it predominantly promotes tumour progression through multifaceted mechanisms, reinforcing its potential as a therapeutic target. In addition, accumulating evidence suggests that SEMA3C contributes to the maintenance of tumour cell stemness during metastatic progression.^33^ SOX4, a stemness‐associated molecule, has been identified as an upstream transcriptional regulator of SEMA3C, while other studies have further shown that SOX4 can also regulate EBF2. These observations are in line with our findings, supporting the notion that SEMA3C may maintain the neural‐related stem‐like differentiation features of tumour cells and thereby further facilitate their interaction with endothelial cells.

FGFs are critical regulators of wound healing and angiogenesis, primarily secreted by endothelial cells, stem cells and damaged cardiac tissue. Among them, FGF‐2 (bFGF) is a potent driver of angiogenesis, orchestrating endothelial migration, invasion and production of plasminogen activators via paracrine mechanisms. In many solid tumours, bFGF is overexpressed and correlates with enhanced metastasis and poor prognosis. These effects are mediated through FGF receptors (FGFR1–4), a family of type I transmembrane tyrosine kinase receptors that activate downstream signalling cascades including RAS–MAPK, PI3K–AKT, STAT and PLCγ pathways.^34^, ^35^, ^36^ Genetic alterations in FGFRs, such as amplification, fusion, rearrangement or kinase domain mutations, can result in constitutive activation, further promoting tumour growth, angiogenesis, invasion and metastasis. Multiple FGFR‐targeted therapies have been developed, including small‐molecule inhibitors (e.g., Erdafitinib, Futibatinib, Infigratinib, Pemigatinib),^37^ antibody–drug conjugates, radiolabelled immunoconjugates, heterobifunctional degraders and CAR‐T cell therapies, with ongoing research aimed at mitigating off‐target toxicity and acquired resistance.^38^, ^39^ Although the HUVEC‐derived TEC model captures key features of endothelial remodelling, future validation using patient‐derived endothelial cells from GCPMs will further strengthen the biological and clinical relevance of our findings.

Transcriptional regulation is central to tumourigenesis, governing gene expression programs that determine cell fate, functional states and adaptive responses. Tumour cells frequently exhibit extensive reprogramming of transcriptional networks, including alterations in chromatin accessibility and enhancer landscapes, driving transitions from normal differentiation to proliferative, invasive and metastatic phenotypes. Transcriptional regulation also profoundly shapes the tumour microenvironment, influencing immune cell infiltration, inflammatory responses and intercellular signalling. Single‐nucleus omics have revealed substantial heterogeneity in transcriptional regulation both within tumours and across patients, correlating with tumour subtype, disease progression and prognosis.^40^ Consequently, dysregulation of transcriptional programs is recognised as a hallmark of cancer, and targeting key transcriptional networks is emerging as a promising strategy for precision oncology.

The early B‐cell factor (EBF) family of transcription factors, comprising EBF1–4, has attracted considerable attention for its context‐dependent roles in cancer. EBFs are critical for maintaining lineage stability, regulating differentiation programs and modulating chromatin architecture and enhancer networks to drive oncogene activation.^41^ Among them, EBF2 has been shown to influence tumourigenesis and energy metabolism, cooperating with SOX4 to control thermogenic gene programs in brown adipocytes,^42^ and can regulate gene expression through epigenetic modifications and miRNA‐mediated pathways. Aberrant EBF2 expression is associated with enhanced tumour cell proliferation, migration and invasion.^43^ Future studies involving larger independent snRNA‐seq cohorts will further strengthen the validation of the identified cell subpopulations and intercellular communication patterns and broaden the applicability of these findings.

In conclusion, our study identifies a tumour cell subpopulation characterised by high SEMA3C expression as a key driver of GCPM. Mechanistically, EBF2 orchestrates transcriptional programs within this subpopulation, upregulating FGF7 and activating FGF7–FGFR2 signalling in VEGFR2‐high TECs. This tumour–endothelial signalling axis promotes vascular remodelling, tumour proliferation and metastatic dissemination. Targeting this pathway may offer a promising therapeutic strategy to disrupt the metastatic microenvironment and inhibit peritoneal dissemination in gastric cancer.

## AUTHOR CONTRIBUTIONS

Yajing Zhou and Weikang Li contributed to conceptualisation. Yajing Zhou, Chao Dong, Pengbo Wang and Caixin Zhu performed data curation and formal analysis. Yajing Zhou, Chao Dong, Weikang Li, Tianzhan Gao and Kaipeng Duan conducted investigation. Yajing Zhou, Chao Dong, Weikang Li, Minghao Sun, Lixing Gu and Tianzhan Gao developed the methodology. Yajing Zhou, Chao Dong, Weikang Li, Qingyu Xie, Lixing Gu and Chengxiang Xu carried out validation. Yajing Zhou, Chao Dong, Weikang Li, Chengxiang Xu and Caixin Zhu performed visualisation. Weikang Li, Minghao Sun, Qingyu Xie and Chengxiang Xu developed software. Yajing Zhou drafted the original manuscript. Yajing Zhou, Chao Dong and Dongbao Li wrote, reviewed and revised the manuscript. Jin Zhou and Dongbao Li acquired funding. Kaipeng Duan and Dongbao Li managed project administration. Kaipeng Duan, Dongbao Li and Jin Zhou supervised the study. Dongbao Li and Jin Zhou provided resources. All the authors read and approved the final manuscript.

## CONFLICT OF INTEREST STATEMENT

The authors declare no conflicts of interest.

## Supporting information



Supporting Information
