# Supplementary material for: Neuro‐associated SEMA3C+ tumour cells promote gastric cancer peritoneal metastasis via EBF2–FGF7–FGFR2‐mediated tumour–endothelial crosstalk
Source: Clin Transl Med. 2026 Oct 15;16(10):e70861. doi: 10.1002/ctm2.70861 (PMC13671878; doi:10.1002/ctm2.70861)
Supplement: Supplementary file 1 [file CTM2-16-e70861-s001.docx]

**Figure S1**

**Fig. S1 a)** GSEA analysis showing positive enrichment of developmental regulation–related signaling pathways in the tumor cell subpopulation. **b)** GSEA analysis showing positive enrichment of metabolic reprogramming–related signaling pathways in the tumor cell subpopulation. **c)** GSEA analysis showing strong activation of invasive remodeling-related signaling pathways in the tumor cell subpopulation. **d)** GSEA analysis showing immune interaction and adhesion suppressed–related signaling pathways in the tumor cell subpopulation.

**Figure S2**

**Fig. S2 a)** Correlation analysis of TPM expression levels between SEMA3C and CD44 genes. **b)** Correlation analysis of TPM expression levels between SEMA3C and BMI1 genes. **c)** Correlation analysis of TPM expression levels between SEMA3C and CAM1 genes. **d)** Correlation analysis of TPM expression levels between SEMA3C and THY1 genes. **e)** Heatmap of branch-dependent genes that are highly expressed along the pseudotime trajectory. **f)** Immunofluorescence images of CD31 and VWF expression in HUVEC cells (scale bar: 100 μm).

**Figure S3**

**Fig. S3 a)** Bright-field images and viability assessment of gastric cancer peritoneal metastasis–derived organoids treated with varying drug concentrations (scale bar: 200 μm). **b)** Violin plot showing the proportion of Ki67-positive cells across different groups. **c)** Violin plot showing FGFR2 fluorescence intensity across experimental groups. **d)** Violin plot showing EBF2 fluorescence intensity across experimental groups.
